# High-throughput multiplex HLA genotyping by next-generation sequencing using multi-locus individual tagging

**DOI:** 10.1186/1471-2164-15-864

**Published:** 2014-10-06

**Authors:** Philip K Ehrenberg, Aviva Geretz, Karen M Baldwin, Richard Apps, Victoria R Polonis, Merlin L Robb, Jerome H Kim, Nelson L Michael, Rasmi Thomas

**Affiliations:** U. S. Military HIV Research Program (MHRP), Walter Reed Army Institute of Research, 503 Robert Grant Avenue, Silver Spring, MD USA; Henry M. Jackson Foundation for the Advancement of Military Medicine, Bethesda, MD USA; LEIDOS Inc, Frederick National Laboratory for Cancer Research, Frederick, MD USA

**Keywords:** HLA, NGS, HLA typing, Illumina MiSeq

## Abstract

**Background:**

Unambiguous human leukocyte antigen (HLA) typing is important in transplant matching and disease association studies. High-resolution HLA typing that is not restricted to the peptide-binding region can decrease HLA allele ambiguities. Cost and technology constraints have hampered high-throughput and efficient high resolution unambiguous HLA typing. We have developed a method for HLA genotyping that preserves the very high-resolution that can be obtained by next-generation sequencing (NGS) but also achieves substantially increased efficiency. Unambiguous HLA-A, B, C and DRB1 genotypes can be determined for 96 individuals in a single run of the Illumina MiSeq.

**Results:**

Long-range amplification of full-length HLA genes from four loci was performed in separate polymerase chain reactions (PCR) using primers and PCR conditions that were optimized to reduce co-amplification of other HLA loci. Amplicons from the four HLA loci of each individual were then pooled and subjected to enzymatic library generation. All four loci of an individual were then tagged with one unique index combination. This multi-locus individual tagging (MIT) method combined with NGS enabled the four loci of 96 individuals to be analyzed in a single 500 cycle sequencing paired-end run of the Illumina-MiSeq. The MIT-NGS method generated sequence reads from the four loci were then discriminated using commercially available NGS HLA typing software. Comparison of the MIT-NGS with Sanger sequence-based HLA typing methods showed that all the ambiguities and discordances between the two methods were due to the accuracy of the MIT-NGS method.

**Conclusions:**

The MIT-NGS method enabled accurate, robust and cost effective simultaneous analyses of four HLA loci per sample and produced 6 or 8-digit high-resolution unambiguous phased HLA typing data from 96 individuals in a single NGS run.

**Electronic supplementary material:**

The online version of this article (doi:10.1186/1471-2164-15-864) contains supplementary material, which is available to authorized users.

## Background

The human leukocyte antigen (HLA) molecules are encoded by genes located within a 3.6 Mb region on chromosome 6p21. The defining feature of HLA genes is their extreme polymorphism (http://www.ebi.ac.uk/ipd/imgt/hla/stats.html). These genes encode surface expressed glycoproteins with a peptide-binding region (PBR) that presents peptides to autologous T lymphocytes and NK cells during the immune surveillance process. HLA molecules thus have a central role in both innate and adaptive immune responses [1, 2] and exert strong influence on cancer, autoimmunity, infectious diseases, and transplantation [3].

HLA genes contain five to eight exons and range in length from 4–17 Kb. Due to cost and time constraints most high-resolution 4-digit HLA genotyping methods employ sequence-based typing (SBT) or differential probe hybridization techniques primarily focused on the PBR consisting of exons 2 and 3 (546 bp total) for class I, and exon 2 (~270 bp) for class II loci. The excluded regions include the other exons, enhancer or promoter regions, introns, and the untranslated regions (UTR). Variation in these regions may be functionally important and can influence HLA transcription rate, gene expression, splice site variation, mRNA stability, and protein structural integrity [4–6]. Also importantly, genotyping when limited to the sequences in PBR may give rise to HLA types that are not phase-defined or are ambiguous.

HLA typing methods based on whole gene sequences extending from enhancer/promoter through 3′UTR regions could address phasing and ambiguity problems that arise due to conventional partial sequencing methods and potentially identify sequences of functional importance that may otherwise be overlooked. The advent of next-generation sequencing (NGS) technologies has facilitated high-throughput parallel sequencing of the human genome and made contiguous sequencing of long genes a reality. Several groups have applied NGS technologies to genotype the highly polymorphic HLA genes using various amplification and library preparation strategies, sequencing platforms, and sequence analysis approaches to greatly enhance sequencing coverage depth and resolve ambiguities [7–14]. Limitations in these reports include relatively small sample sizes, non-contiguous amplicons, partial gene sequencing, multiple PCR for one locus, and use of laborious library preparation and complex sequence analysis methods.

Hosomichi et. al. simplified the laboratory component of NGS HLA typing technology by using a commercial transposase-based kit for fragmentation and library preparation of long-range contiguous sequences of individual HLA genes, which were then sequenced on the Illumina MiSeq platform [10]. This method is however limited to analysis of four HLA loci in 24 individuals per sequencing run due to their utilization of a unique index tag for each locus from an individual. Since multiple HLA loci genotypes are typically required from each individual we developed a multiplex individual tagging approach to enable HLA-A, B, C, and DRB1 to be simultaneously typed in each of the 96 homozygous or heterozygous individuals per sequencing run. Contiguous highly locus-specific whole gene PCR amplifications of four HLA loci per individual were pooled and subjected to library construction using the transposase-based Nextera XT kit (Illumina) and 96 indices. One unique index combination is used collectively for the four loci of each individual and not per locus. The 96 separately indexed multiplex samples, each composed of four loci, are then collectively pooled and sequenced in a single run on the Illumina MiSeq platform. For each individual the four loci either homozygous or heterozygous are discriminated using commercially available NGS HLA typing software. This robust, highly specific, and cost effective approach simultaneously sequences full-length HLA-A, B, C, and DRB1 loci using multi-locus individual tagging and NGS (MIT-NGS). As these four loci are the most polymorphic loci described in the IMGT/HLA database their genotyping by MIT-NGS demonstrates the ability of this method to delineate multiple alleles of very complex loci.

## Methods

### Samples

Fifteen well-characterized HLA typed control genomic DNA (gDNA) samples from HLA Reference Panels were obtained from the International Histocompatibility Working Group (IHWG) (Research Cell Bank, Fred Hutchinson Cancer Research Center, Seattle, WA). Additionally, peripheral blood mononuclear cells (PBMCs) from 81 healthy donors of mainly Asian, Caucasian and African ancestry enrolled in the U.S. Military HIV Research Program (MHRP) RV144 and RV229 study protocols were included in this study. The RV144 study was approved by the Human Subjects Research Review Board, Ft Detrick, MD, USA as well as the Thai Ministry of Public Health Ethical Committee, the Mahidol University IRB, and the Royal Thai Army IRB. The RV229 protocol was reviewed and approved by the Walter Reed Army Institute of Research IRB. All individuals gave informed consent for participation in this study. Genomic DNA from the PBMCs was extracted and purified using the QIAamp DNA Blood Mini Kit (Qiagen, Valencia, CA) according to the manufacturers’ suggestions.

### HLA genotyping by Sanger sequence-based typing (SBT)

HLA class I genotyping was performed using the 13th International Histocompatibility Workshop SBT method adapted by Lazaro et al. [15]. HLA-DRB1 genotyping was performed with initial group specific allele amplification using primers that were tagged with the M13 sequence (Additional files 1 and 2) [16–18]. The DRB1 PCR conditions were modified to use a SYBR Green assay consisting of 1X Platinum SYBR Green qPCR Supermix-UDG with ROX (Life Technologies, Carlsbad, CA), 2 ul of the respective 0.625 or 1.25 uM primer mix, and 5 ng of gDNA in a total reaction volume of 5 ul. PCR amplifications were performed in Veriti 384-well thermal cyclers (Life Technologies) using the following parameters: 95C, 3 min; 5 cycles of 95C, 5 sec; 65C, 15 sec; 72C, 30 sec; 26 cycles of 95C, 30 sec; 58C, 15 sec; 72C, 30 sec; 5 cycles of 95C, 5 sec; 56C, 30 sec; 72C, 1 min; followed by a 72C, 7 min extension. An amplified product was detected by dissociation curve analyses using a 7900HT Fast Real-Time PCR System with SDS v2.4 software (both Life Technologies) [19] and purified using 1.8X Agencourt AMPure beads on a Biomek NXp instrument (both Beckman Coulter, Miami, FL). Purified amplicons were sequenced with M13 forward and reverse primers using BigDye Terminator cycle sequencing v3.1 kits and a 3730xl DNA analyzer (both Life Technologies). HLA sequences from all four loci were analyzed using the Assign ATF software v1.0.2.45 (Conexio Genomics, Fremantle, Australia).

### Long-range PCR template preparation

Previously described locus specific long-range PCR primers (Additional file 3) were used to amplify HLA-A, B, and C genes consisting of 5466, 4609, and 4802 base pairs, respectively [9]. A modified version of the Hosomichi et al. primers (Additional file 3) was used for HLA-DRB1 amplification to minimize allelic imbalance and dropout [10]. Four of the IHWG reference samples (IHW09021, IHW0924, IHW09056, IHW09381) were duplicates and were used to evaluate consistency of the MIT-NGS method across 96-well plates. The original PCR amplification conditions for all loci were modified. Briefly HLA-A, B, and C loci were amplified in 10 ul reaction volumes consisting of 25 ng gDNA, 1X Buffer, 2 mM MgSO_4_, 200 uM dNTPs (Fermentas, Pittsburgh, PA), 5% DMSO (Sigma-Aldrich, St. Louis, MO), 200 nM of the respective HLA locus-specific primer mix, and 0.5 U Platinum Taq HiFi (Life Technologies). The HLA-DRB1 locus was amplified in 10 ul reaction volumes consisting of 25 ng gDNA, 1X GXL Buffer, 200 uM dNTPs, 200 nM of the respective primer mix (Sigma-Genosys), and 0.25 U PrimeSTAR GXL (Takara Bio Inc, Japan). PCR cycling parameters are shown in Additional file 4. To confirm PCR amplification, 1 ul of each PCR product was screened for the presence of amplicon on 1% agarose gels. The remaining product was purified using Agencourt AMPure beads on a Biomek NXp instrument (both Beckman Coulter). The purified product was then quantitated with the Qubit Fluorometric system (Life Technologies).

### Multiplex library preparation and next-generation sequencing

Each of the four purified HLA amplicons from an individual were diluted in a single well of a 96-well plate to a collective concentration of 0.16 ng/ul. Library construction including simultaneous transposase-mediated fragmentation and adaptor addition was performed with the Nextera XT DNA sample preparation kit (Illumina, San Diego, CA). Briefly multiplex sample wells containing 3.2 ng (0.8 ng of each amplicon) were dual-indexed, bead-normalized, and pooled. Four loci from each of the 96 individuals were tagged with an identical individual specific index pair, and thus 96-paired indices could be used to tag 768 alleles (96 individuals x 4 loci x 2 alleles). The resulting pooled sequencing library was diluted in HT-1/5% Tris–HCl, pH7.5 [20] and sequenced on a MiSeq instrument using the paired-end 500 cycle (2 x 250 bp paired-end) MiSeq Reagent Kit (both Illumina).

Samples with MIT-NGS genotypes that were discordant with the SBT method were repeated by increasing the DRB1 locus amplicon product input to three-fold relative to the class I loci to account for the average three-fold amplicon size difference. Purified HLA amplicons from an individual were diluted in a single well of a 96-well plate to concentrations of 0.16 ng/ul for the HLA class I loci and 0.48 ng/ul for DRB1. During the library preparation, multiplex sample wells containing 4.8 ng (0.8 ng of each HLA class I amplicon and 2.4 ng of the -DRB1 amplicon) were dual-indexed, bead-normalized, and pooled. Samples were then run on the MiSeq as described above.

### Data analysis

MiSeq Reporter analysis software (Illumina) generated FASTQ sequence reads and used a 1.3 Mb reference extending from HLA-DPB1 through HLA-A of the hg19 build to create BAM alignments. The Integrative Genomics Viewer (IGV) browser was used to independently review sequence alignments generated by the Miseq Reporter [21]. HLA genotyping calls based on exonic sequences were made using Omixon Target software v1.7 (Omixon Biocomputing Kft, Budapest, HU). Version 1.8 of the Omixon Target software (Omixon) was used to reanalyze the HLA locus discordance(s). The software used reference sequences from the IMGT/HLA database Release v3.10.0 and v3.15.0. Raw Sequence data was submitted to the European Nucleotide Archive repository, (Study Accession number PRJEB7337) and compiled in the European Molecular Biology Laboratory (EMBL/EBI) Nucleotide Sequence Database (http://www.ebi.ac.uk/ena/data/view/PRJEB7337). HLA ambiguities from the SBT HLA-typing method were reported using the G group nomenclature (http://hla.alleles.org/alleles/g_groups.html).

## Results

### Long-range HLA amplifications

Full-length HLA-A, B, and C loci were contiguously amplified using long-range PCR. These class I PCR parameters were optimized to minimize allele bias and increase locus specificity. The full-length class II HLA-DRB1 locus was contiguously amplified using modified primers and PCR conditions to facilitate amplification of all DRB1 alleles, ranging from 10.8-17.1 kb and reduce co-amplification of other class II genes [22]. Representative purified long-range PCR amplicons from the four loci of one sample are shown in Additional file 5.

### Full-length multiplex sequencing

We used a novel method, MIT-NGS that combined long-range PCR and Illumina MiSeq-based multiplex sequencing for HLA typing of four loci of 96 individuals in a single run. Quality scores ≥ Q30 for 88.9% of the reads were obtained, with 91.7% of the 17.111 million clusters passing the quality filter that eliminates reads having more than one base call with chastity scores of <0.6 in the first 25 cycles. Poor quality reads prevented analyses of all loci for one of the 96 individuals. Thus, of the initial 768 sequenced alleles (96 individuals × 4 loci × 2 alleles) 760 were further analyzed for HLA genotypes. Omixon was used to analyze the FASTQ files and generate HLA typing calls. Sequence metrics from the MiSeq-Omixon pipeline are summarized in Table 1.

**Table 1:** **Summary of the NGS HLA Omixon software sequencing metrics from the MiSeq run**

Locus	Average depth of all exons^a^	Allele ratios^b^	Detection <100% of all exons^c^
A	1311 (87.4-2580)	0.7-1.25	38 (96.2-99.9%)
B	1192 (104–2820)	0.78-1.24	112 (92.3-99.9%)
C	1527 (94–3570)	0.59-1.76	114 (98.8-99.9%)
DRB1	475 (26.8-1520)	0.35-3.02	5 (92.9-95.4%)

^a^Average exonic sequence read depth per base pair.

^b^Ratio of average sequence read coverage of alleles one and two.

^c^Number of alleles with less than 100% sequence coverage.

The range in sequence coverage is indicated in parentheses.

### HLA genotyping ambiguities

The HLA-SBT typing method generates genotypes based solely on the highly polymorphic PBR defined by exons 2–3 for class I, and exon 2 for class II. HLA alleles that share sequence identity over these regions but differ elsewhere in the sequence are considered ambiguous alleles. HLA genotypes obtained by HLA-SBT were compared to those from the novel MIT-NGS method (Additional file 6). We identified 13 different ambiguous HLA alleles, with 50 occurrences in 41 individuals, emphasizing the potential of full-length sequencing to enhance resolution and typing accuracies (Table 2). Ambiguities were detected in at least one locus in 43.2% (41 of 95) of sequenced individuals and 6.6% (50 of 760) of typed alleles. Four of these individuals had ambiguities at two or three different loci. The MIT-NGS method was able to differentiate HLA-C*07:01:01G allele into three allele subtypes, namely 07:06, 07:18, and 07:01:02. The most frequent SBT/MIT-NGS allele ambiguities at each locus were HLA-A*23:01:01G/23:17 (2 of 2); HLA-B*40:01:01G/40:01:02 (7 of 12); HLA-C*07:01:01G/07:06 (8 of 29), and HLA-DRB1*14:01:01G/14:54:01 (6 of 7). Importantly, 25 of the 50 ambiguities were due to non-synonymous protein coding changes. All of the allele ambiguities except one resulted from differences at one or two polymorphic SNP positions. An exception was the C*04:82 allele which had a 9 bp insertion relative to *04:01:01 in exon 5 at position 970. The MIT-NGS method was able to resolve all HLA allele ambiguities that were observed in the SBT method.

**Table 2:** **HLA ambiguities between SBT and MIT-NGS HLA genotyping methods**

Locus	SBT	Alleles in G group*	MIT-NGS
A	23:01:01G	7	23:17 (2)
B	40:01:01G	17	40:01:02 (7)
	07:05:01G	2	07:06 (3)
	35:01:01G	18	35:01:23 (2)
C	07:01:01G	13	07:06 (8), 07:18 (4), 07:01:02 (1)
	03:02:01G	10	03:02:02 (7)
	12:02:01G	3	12:02:02 (7)
	04:01:01G	20	04:82 (1)
	15:05:01G	4	15:05:02 (1)
DRB1	14:01:01G	2	14:54 (6)
	11:06:01G	2	11:129 (1)

The number of individuals with this allele is shown in parentheses.

*Number of ambiguous alleles defined by the G group (IMGT/HLA release 3.16.0).

### HLA typing discordances

We evaluated concordance between the novel MIT-NGS method and the SBT method for all 95 of the individuals analyzed. The MIT-NGS and SBT genotype concordance rates were 100% for HLA-A (190 of 190), 98.9% for HLA-B (188 of 190), 100% for HLA-C (190 of 190), and 94.2% for HLA-DRB1 (179 of 190). These apparent discordances in 13 alleles were due to miscalls or ambiguous allele combinations and are summarized in Table 3. Closer examination of alignments using reference sequences from the IMGT/HLA database and the IGV browser showed that the MIT-NGS calls were correct rather than the original SBT results. Seven discordances were due to the mis-incorporation of a non-complementary base of an SBT primer during PCR amplification. Six discordances resulted from ambiguous allele combinations that occurred when sequences of paired alleles shared respective group specific differentiating SNP(s) that could not be differentiated by SBT and remained unphased.

**Table 3:** **HLA discordances between SBT and MIT-NGS HLA genotyping methods**

Locus	SBT	MIT-NGS	Classification
B	14:03/35:01:01G	**14:02:01/35:08:01** (1)	ambiguous allele combination
DRB1	01:02:01/08:01:05	01:02:01/**08:01:03** (1)	nucleotide mismatch
	08:01:05/08:01:05	**08:01:03/08:01:03** (1)	nucleotide mismatch
	10:01:01/11:01:01G	10:01:01/**11:01:02** (1)	nucleotide mismatch
	03:01:01G/11:01:01G	03:01:01/**11:01:02** (1)	nucleotide mismatch
	11:01:01G/13:01:01G	**11:01:02**/13:01:01 (1)	nucleotide mismatch
	11:01:01G/15:03:01G	**11:01:02**/15:03:01 (1)	nucleotide mismatch
	13:01:01/14:07:01	**13:02:01/14:54:01** (2)	ambiguous allele combination

Discordant alleles are highlighted in bold.

The number of individuals with a given genotype is indicated in parentheses.

The results reported above do not include four HLA-DRB1 discordances that were not adequately detected by MIT-NGS initially. These samples had allele dropout relative to the SBT calls and were repeated using a three-fold increase in the DRB1 amplicon concentration ratio compared to the class I loci. The resulting calls from MIT-NGS matched the HLA-SBT genotypes (Additional file 7). Overall, the MIT-NGS method generated very high-resolution HLA typing for 95 individuals and accurately genotyped 100% of the HLA class I and DRB1 locus alleles.

## Discussion

Successful HLA genotyping depends on robust amplification strategies, high quality sequencing output, and accurate sequence analysis algorithms. Recent reports have demonstrated the feasibility of applying long-range PCR based approaches to generate 6 or 8-digit HLA typing using different NGS platforms [9, 10]. The analysis process ranged from direct local alignment searches of the IMGT/HLA database for nearest matched alleles [9]; to local alignment searches after phase conferring heterozygous SNP mapping and alignment against hg19 human genome reference sequences [10]. To further increase throughput, target specificity, and cost effectiveness we developed a novel genotyping method called MIT-NGS that uses highly specific primer and PCR conditions to amplify full-length HLA-A, B, C and DRB1 loci from 96 individuals. Pooled amplicons from four loci of each individual were tagged with the same unique index combination. This multiplex individual tagging method enabled simultaneous indexing of all 384 loci with 96 paired indices. All indexed samples were then pooled and sequenced on a single run of the Illumina MiSeq platform.

To decrease complexity associated with NGS sequence analyses, FASTQ files from the MiSeq instrument were directly analyzed by a commercially available NGS HLA typing analysis software. Omixon has alignment algorithms that map NGS short reads to allele sequences in the IMGT/HLA database. Since many alleles in the database lack intronic sequences, analysis that includes only exonic sequences currently provides the most consistent HLA typing results. Reads are mapped to multiple alleles with limited mismatches, and the resulting allele candidates are compared statistically for the depth and evenness of coverage. Allele depth percentages were greater than 25% for minor alleles of heterozygous samples for all four loci, with the majority greater than 40% (Additional file 6). Average sequence read coverage percentages across exons for all samples were greater than 90% (Additional file 8).

**Figure 1:**
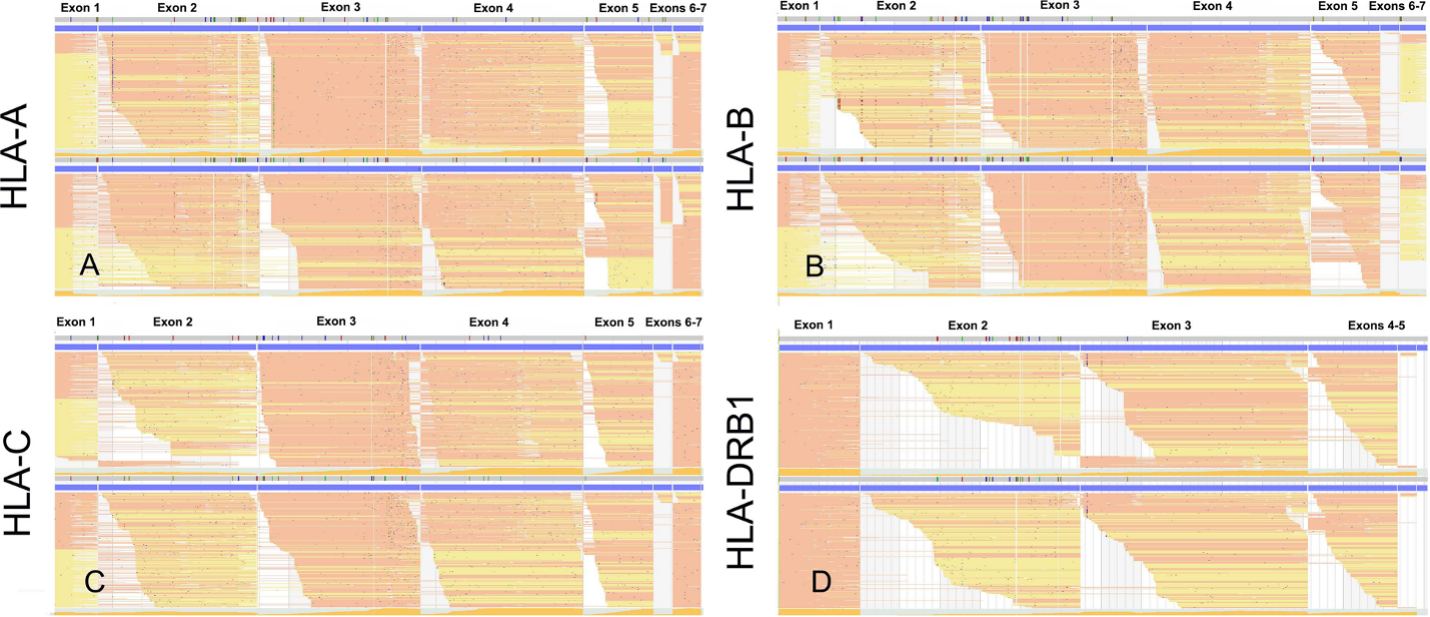
**Short read pile-ups of HLA sequences.** A graphical representation of sequence read alignments for **(A)** HLA-A, **(B)** HLA-B, **(C)** HLA-C, and **(D)** HLA-DRB1 paired alleles compiled by the Omixon software from MiSeq generated FASTQ files. Only exons are shown in this alignment for simplicity.

The SBT method targets the highly polymorphic PBR, encoded by one or two exons, and relies on interpretation of differences in chromatogram traces at putative SNP positions. In contrast, MIT-NGS generates genotypes from full-length sequences encompassing all exons, based on allele sequence availability in the IMGT/HLA database. Short read pileups of each allele with corresponding reads can be reviewed visually in the Omixon software facilitating quality checks and validation of alternate allele options (Figure 1). Vast sequencing depth and coverage also enables generation of high-resolution and phased genotypes. In comparisons between the SBT and MIT-NGS methods, 50 ambiguous alleles were observed. MIT-NGS consistently produced the higher resolution non-ambiguous HLA allele calls, predominantly due to sequence coverage of all polymorphic positions in regions not restricted to the PBR. Further, half of all the ambiguous calls were due to variation that impacted the HLA protein, highlighting the importance of the MIT-NGS method. A recent study demonstrating HLA typing using the NGS Illumina MiSeq platform was limited to exons 2–3 and hence did not address these ambiguities [8]. Enhanced resolution unambiguous HLA typing is particularly relevant in solid organ and hematopoietic stem cell transplantation where closely related alleles may associate with very different outcomes [23, 24].

The combined rates of concordance including alleles ambiguously typed by SBT but resolved by MIT-NGS as concordant for all four HLA loci between the two methods was 98.3%. The remaining genotype discordances between the MIT-NGS and SBT methods were due to the accuracy of the MIT-NGS calls compared to the SBT HLA typing. Even though HLA-SBT is the gold standard for HLA-typing this method was susceptible to undefined phasing as well as nucleotide misincorporations introduced by an HLA-DRB1 reverse primer in exon 2. We established that the MIT-NGS method was accurate because of its ability to phase as well as being able to visualize the correct polymorphism with high sequence depth and coverage in key exons. In the initial run of the MIT-NGS method, four miscalls occurred at the DRB1 locus due to allelic imbalance and dropout. Since the average class I and DRB1 amplicon sizes are ~5 and 17 kb, respectively, we increased the DRB1 amplicon input by ~ three-fold to enable equimolar amplicon representation for these samples. This approach corrected the miscalls observed in the HLA-DRB1 loci and increased the DRB1 MIT-NGS HLA typing success rate to 100%. Overall this also improved the average coverage and depth of all four loci (Additional file 7).

The ability of the MIT-NGS method to capture the extensive HLA diversity in different populations [25–27] was highlighted by genotyping ethnically diverse individuals. This method successfully genotyped 33 HLA-A, 55 HLA-B, 30 HLA-C and 41 HLA-DRB1 alleles from 95 individuals belonging to major ethnic groups including Asian (47%), Caucasian (26%), and African (17%) ancestry. Furthermore, this method was robust enough to identify several alleles that were not detected by PCR-SBT previously and were present at very low frequencies such as, A*02:17:01, A*02:20:02, A*23:17, B*35:43:01, B*39:08, C*02:10, C*04:52, C*04:82, C*07:18, DRB1*03:15, DRB1*04:87 and DRB1*11:129. Other important challenges that need to be addressed for high-throughput HLA genotyping include cost effectiveness, ease of setup, hands-on laboratory time, and sequence analyses tools. We have established that the laboratory costs associated with the four loci multiplex MiSeq-based MIT-NGS HLA typing method for 96 individuals are considerably less than those incurred with SBT typing. Similarly, PCR setup and downstream sequencing processes for the MIT-NGS method are comparable to the SBT method, but less laborious and time consuming since sequencing reactions are reduced due to multiplexing and pooling (Additional file 9). Though we used commercially available software for HLA sequence analyses, the MIT-NGS method is flexible enough to be used with either in-house or other NGS HLA typing software. Moreover, this multiplex method can also be adapted to sequence other genes.

## Conclusions

The novel MIT-NGS method for HLA typing comprising multiplex full-length HLA sequencing and analysis proved to be an efficient and accurate tool for very high-resolution HLA genotyping. Compared to the SBT method, MIT-NGS concordantly called all HLA alleles and was further able to clarify and resolve ambiguities. This method can be used for research and with further work we envisage that HLA typing by NGS will improve transplantation matching, because of the increased resolution demonstrated here. The current method types the four most diverse HLA loci: HLA-A, B, C and DRB1. With next-generation sequencing and analysis technologies improving rapidly it will be possible to expand the multi-locus individual tagging multiplex approach described here to other HLA class II loci and increase throughput. The potential to genotype 96 samples for four of the most variable HLA loci, at a cost comparable to Sanger SBT methods but with the increased resolution defined by NGS, is a major advance with clear benefit to the HLA community. The ability of MIT-NGS to accurately, robustly, efficiently, and cost effectively genotype HLA across ethnically diverse populations has important implications for disease studies, vaccine trials, and transplantation science.

## Electronic supplementary material

Additional file 1: **HLA-DRB1 SBT amplification primers.** (DOCX 95 KB)

Additional file 2: **HLA-DRB1 group specific primer combinations and allele specificities.** (DOCX 69 KB)

Additional file 3: **Long-range amplifying primers for MIT-NGS.** (DOCX 90 KB)

Additional file 4: **Long-range amplification cycling parameters for all four loci.** (DOCX 61 KB)

Additional file 5: **Long-range PCR HLA amplicons.** (DOCX 174 KB)

Additional file 6: **MIT-NGS allele calls and analysis metrics for 96 individuals.** (XLSX 89 KB)

Additional file 7: **MIT-NGS allele calls and analysis metrics for the initial DRB1 discordant samples.** (XLSX 12 KB)

Additional file 8: **Average sequence read coverage percentage of exons across HLA alleles.** (DOCX 141 KB)

Additional file 9: **Comparison of the SBT and MIT-NGS HLA genotyping laboratory methods.** (DOCX 76 KB)
