# Supplementary material for: High-throughput multiplex HLA genotyping by next-generation sequencing using multi-locus individual tagging
Source: BMC Genomics. 2014 Oct 6;15(1):864. doi: 10.1186/1471-2164-15-864 (PMC4196003; doi:10.1186/1471-2164-15-864)
Supplement: Supplementary file 1 — Additional file 1: HLA-DRB1 SBT amplification primers. (DOCX 95 KB) [file 12864_2014_6530_MOESM1_ESM.docx]

**Additional File 1 HLA-DRB1 SBT amplification primers**

| **Primer** | **Orientation** | **Sequence** | **Reference** |
| --- | --- | --- | --- |
| RBAMP-1 | Forward | **TGT AAA ACG ACG GCC AGT G**TT CTT GTG GCA GCT TAA GTT | [[17](#_ENREF_17)] |
| RBAMP-2 | Forward | **TGT AAA ACG ACG GCC AGT G**TT CCT GTG GCA GCC TAA GAG G | [[17](#_ENREF_17)] |
| RB52 | Forward | **TGT AAA ACG ACG GCC AGT G**GT TTC TTG GAG TAC TCT ACG TC | [[18](#_ENREF_18)] |
| RBAMP-4 | Forward | **TGT AAA ACG ACG GCC AGT G**GT TTC TTG GAG CAG GTT AAA C | [[17](#_ENREF_17)] |
| RBAMP-7A | Forward | **TGT AAA ACG ACG GCC AGT G**CA CGT TTC CTG TGG CAG GG | [[16](#_ENREF_16)] |
| RB128 | Forward | **TGT AAA ACG ACG GCC AGT G**GT TTC TTG GAG TAC TCT ACG GG | [[17](#_ENREF_17)] |
| RBAMP-10 | Forward | **TGT AAA ACG ACG GCC AGT G**CG GTT GCT GGA AAG ACG CG | [[18](#_ENREF_18)] |
| RBAMP-9B | Forward | **TGT AAA ACG ACG GCC AGT G**GT TTC TTG AAG CAG GAT AAG TTT | [[18](#_ENREF_18)] |
| RBAMP-B | Reverse | **CAG GAA ACA GCT ATG ACC ATG** CCG CTG CAC TGT GAA GCT CT | [[17](#_ENREF_17)] |
| 7.9-3 | Reverse | **CAG GAA ACA GCT ATG ACC ATG** CCC GTA GTT GTG TCT GCA CAC | [[18](#_ENREF_18)] |

M13 sequences are indicated in bold
