# Supplementary material for: High-throughput multiplex HLA genotyping by next-generation sequencing using multi-locus individual tagging
Source: BMC Genomics. 2014 Oct 6;15(1):864. doi: 10.1186/1471-2164-15-864 (PMC4196003; doi:10.1186/1471-2164-15-864)
Supplement: Supplementary file 2 — Additional file 2: HLA-DRB1 group specific primer combinations and allele specificities. (DOCX 69 KB) [file 12864_2014_6530_MOESM2_ESM.docx]

**Additional File 2 HLA-DRB1 group specific primer combinations and allele specificities**

| **Primer mix** | **DRB1 alleles** | **Forward primer** | **Reverse primer** | **Mix concentration (uM)** | **Amplicon size (bp)*** |
| --- | --- | --- | --- | --- | --- |
| 01 | 01 | RBAMP-1 | RBAMP-B | 0.625 | 301 |
| 03 | 03, 11, 13, 14 | RB52 | RBAMP-B | 0.625 | 303 |
| 04 | 04, 14:10 | RBAMP-4 | RBAMP-B | 0.625 | 303 |
| 07 | 07 | RBAMP-7A | RBAMP-B | 0.625 | 306 |
| 08 | 08, 12, 14:04 | RB128 | RBAMP-B | 0.625 | 303 |
| 09 | 09 | RBAMP-9B | 7.9-3 | 1.25 | 276 |
| 10 | 10 | RBAMP-10 | RBAMP-B | 0.625 | 247 |
| 15 | 15, 16 | RBAMP-2 | RBAMP-B | 0.625 | 301 |

* Amplicon size includes M13 sequence
