# Supplementary material for: High-throughput multiplex HLA genotyping by next-generation sequencing using multi-locus individual tagging
Source: BMC Genomics. 2014 Oct 6;15(1):864. doi: 10.1186/1471-2164-15-864 (PMC4196003; doi:10.1186/1471-2164-15-864)
Supplement: Supplementary file 3 — Additional file 3: Long-range amplifying primers for MIT-NGS. (DOCX 90 KB) [file 12864_2014_6530_MOESM3_ESM.docx]

**Additional File 3 Long-range amplifying primers for MIT-NGS**

| **Locus** | **Primer mix** | **Sequence** | **Reference** |
| --- | --- | --- | --- |
| A | A F1 | AAC TCA GAG CTA AGG AAT GAT GGC AAA T | [[9](#_ENREF_9)] |
|  | A F2 | AAC TCA GAG CTA TGG AAT GAT GGT AAA T | [[9](#_ENREF_9)] |
|  | A R1 | ATA TAA CCA TCA TCG TGT CCC AAG GTT C | [[9](#_ENREF_9)] |
| B | B F1 | CCC GGT TGC AAT AGA CAG TAA CAA A | [[9](#_ENREF_9)] |
|  | B R1 | GGG TCC AAT TTC ACA GAC AAA TGT | [[9](#_ENREF_9)] |
| C | C F1 | TGC TTA GAT GTG CAT AGT TCA CGA A | [[9](#_ENREF_9)] |
|  | C F2 | TGC TTA GAT GTG CAT AGT TCC GGA A | [[9](#_ENREF_9)] |
|  | C R1 | TGG ACC CAA TTT TAC AAA CAA ATA | [[9](#_ENREF_9)] |
| DRB1 | DRB1 F1.1 | GCA TCC ACA GAA TCA CAT TTT CTA GTG TT | [[10](#_ENREF_10)]* |
|  | DRB1 F1.2.1 | TCC ACA GAA TCA CAG CAT TTT CTA GTG TT |  |
|  | DRB1 F1.3 | GCA TCC ACA GAA TCA CAT TTT CCA GTA TT |  |
|  | DRB1 F1.4.1 | TCC ACA GAA TCA CAG CAT TTT CCA GTA TT |  |
|  | DRB1 R2.1 | TGA TTG ACT TGC TGG CTG GTT TCT CAT C | [[10](#_ENREF_10)]* |

F - forward primer; R - reverse primer

* Forward and reverse primer nomenclature from Hosomichi et al. was inter-changed to reflect relative gene orientation
