# Supplementary material for: High-throughput multiplex HLA genotyping by next-generation sequencing using multi-locus individual tagging
Source: BMC Genomics. 2014 Oct 6;15(1):864. doi: 10.1186/1471-2164-15-864 (PMC4196003; doi:10.1186/1471-2164-15-864)
Supplement: Supplementary file 4 — Additional file 4: Long-range amplification cycling parameters for all four loci. (DOCX 61 KB) [file 12864_2014_6530_MOESM4_ESM.docx]

**Additional File 4 Long-range amplification cycling parameters for all four loci**

| **Locus** | **Length (kb)** | **Enzyme** | **Cycles** | **PCR parameters*** |
| --- | --- | --- | --- | --- |
| A | 5.466 | PT | 35 | 94C, 30s; 55C, 45s; 68C, 5.5m |
| B | 4.609 | PT | 35 | 94C, 30s; 60C, 45s; 68C, 5.5m |
| C | 4.802 | PT | 35 | 94C, 30s; 55C, 45s; 68C, 5.5m |
| DRB1 | 10.8-17.1 | PS | 30 | 98C, 10s; 58C, 15s; 68C, 10m |

PT - Platinum Taq HiFi; PS - PrimeSTAR GXL; s - second; m - minute

* All cycling parameters were both preceded by a 94C, 2m initial denaturation step; and followed by a 68C, 10m final extension step
