# Supplementary material for: High-throughput multiplex HLA genotyping by next-generation sequencing using multi-locus individual tagging
Source: BMC Genomics. 2014 Oct 6;15(1):864. doi: 10.1186/1471-2164-15-864 (PMC4196003; doi:10.1186/1471-2164-15-864)

**Additional File 5 Long-range PCR HLA amplicons.** Purified PCR products amplified from HLA-A (5.4 kb), B (4.6 kb), C (4.8 kb), and DRB1 (10.8-17.1 kb) loci from a representative sample were electrophoresed through a 1% agarose gel in lanes 2-5, respectively. Lanes 1 and 6 contains combined molecular weight markers consisting of ΦX174/Hae III and λ/HindIII.


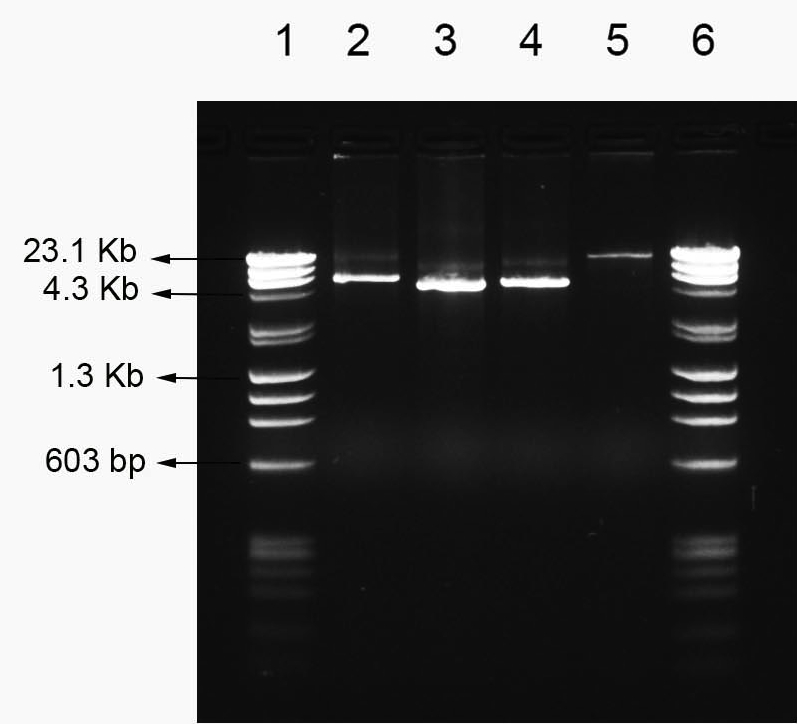

Supplement: Supplementary file 5 — Additional file 5: Long-range PCR HLA amplicons. (DOCX 174 KB) [file 12864_2014_6530_MOESM5_ESM.docx]
