# Supplementary material for: High-throughput multiplex HLA genotyping by next-generation sequencing using multi-locus individual tagging
Source: BMC Genomics. 2014 Oct 6;15(1):864. doi: 10.1186/1471-2164-15-864 (PMC4196003; doi:10.1186/1471-2164-15-864)

**Additional File 8 Average sequence read coverage percentage of exons across HLA alleles.** Box and whisker plot showing the average sequence read coverage percentage of exons across all alleles for the four HLA-loci.


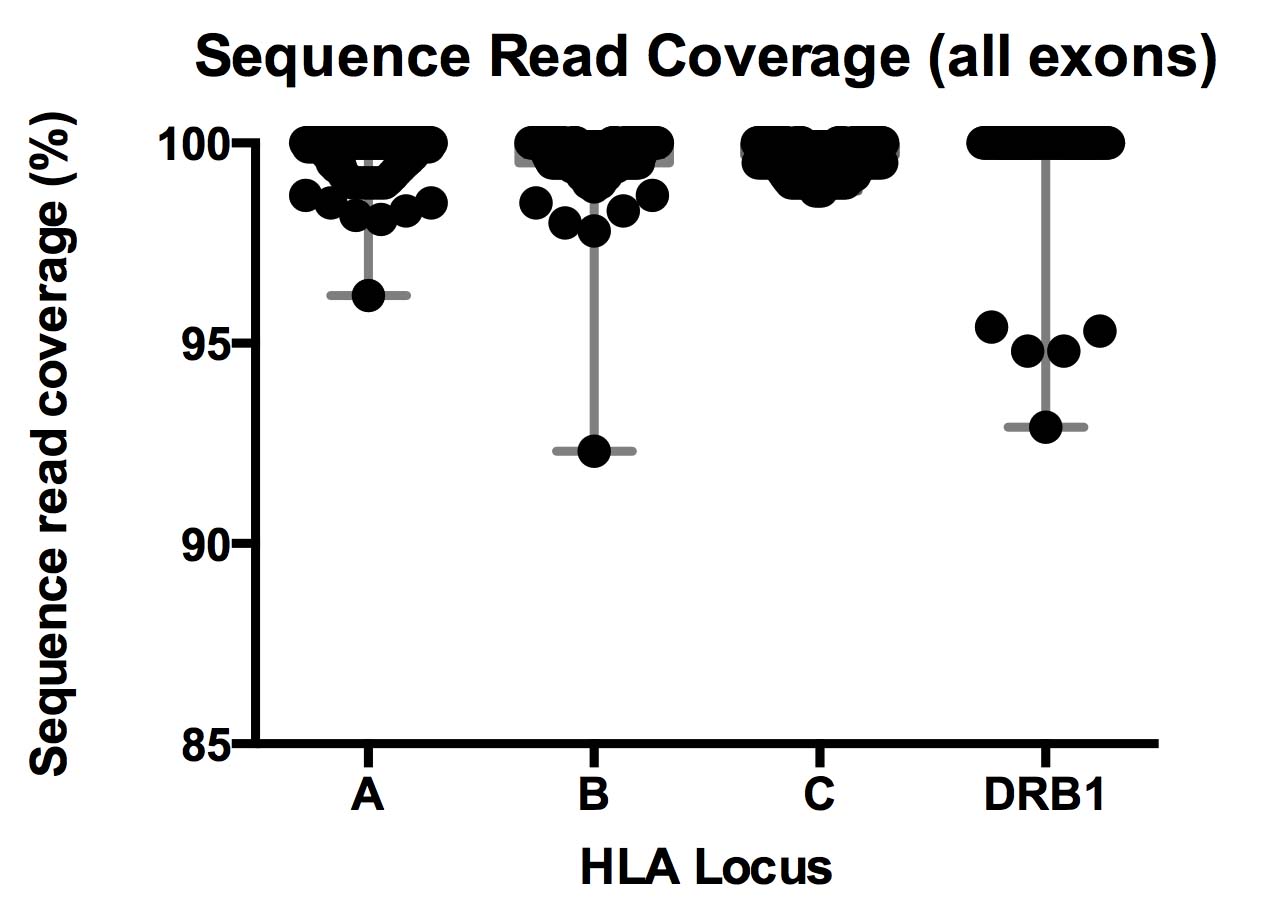

Supplement: Supplementary file 8 — Additional file 8: Average sequence read coverage percentage of exons across HLA alleles. (DOCX 141 KB) [file 12864_2014_6530_MOESM8_ESM.docx]
