# Supplementary material for: High-throughput multiplex HLA genotyping by next-generation sequencing using multi-locus individual tagging
Source: BMC Genomics. 2014 Oct 6;15(1):864. doi: 10.1186/1471-2164-15-864 (PMC4196003; doi:10.1186/1471-2164-15-864)
Supplement: Supplementary file 9 — Additional file 9: Comparison of the SBT and MIT-NGS HLA genotyping laboratory methods. (DOCX 76 KB) [file 12864_2014_6530_MOESM9_ESM.docx]

**Additional File 9 Comparison of the SBT and MIT-NGS HLA genotyping methods**

| **Step** | **SBT** | **MIT-NGS** |
| --- | --- | --- |
| PCR Amplification | 4*96-well plates (exon 2 or 2-3) | 4*96-well plates (whole gene) |
| Quantitation | N/A | Qubit 4*96 well-plates (1/locus) |
| Locus-product dilution | N/A | 4*96-well plates to 2 ng/ul |
| Multiplex dilution | N/A | 1*96-well plate (4 pooled loci) |
| Library preparation | N/A | Nextera XT |
| Dissociation curve analysis | 2*384-well plates | N/A |
| Sequencing PCR | 16*96-well plates (4 loci x 4 primers) | N/A |
| Post-Sequencing PCR clean-up | 16*96-well plates | N/A |
| Sequencing | 16*96 well plates (Capillary 3730xl) | 1*96-well plate (NGS MiSeq) |
| **Total Time (hours)** | **~84** | **~73** |

This table compares the differential procedures and/or time requirements (inclusive of set-up and run times) on a per plate basis for each method. Equivalent procedures between the two methods, such as AMPure clean-up and gel confirmations, are not shown for simplicity.

N/A - not applicable
